# Exploring the acceptability of Option B plus among HIV-positive Nigerian women engaged and not engaged in the prevention of mother-to-child transmission of HIV cascade: a qualitative study

**DOI:** 10.1080/17290376.2018.1527245

**Published:** 2018-09-25

**Authors:** Salome C. Erekaha, Llewellyn J. Cornelius, Melissa L. Bessaha, Abdulmumin Ibrahim, Gabriel D. Adeyemo, Mofoluwake Fadare, Manhattan Charurat, Echezona E. Ezeanolue, Nadia A. Sam-Agudu

**Affiliations:** a International Research Center of Excellence, Institute of Human Virology Nigeria, Abuja, Nigeria; b School of Social Work and College of Public Health, University of Georgia Athens, USA; c School of Social Welfare, Health Sciences Center, Stony Brook, NY, USA; d Faculty of Basic Medical Sciences, College of Health Sciences, University of Ilorin, Ilorin, Nigeria; e Faculty of Health Sciences, Department of Human Biology, University of Cape Town, Cape Town, South Africa; f United Nations Population Fund, Abuja, Nigeria; g Mercy Corps Nigeria, Abuja, Nigeria; h Department of Epidemiology and Public Health and Director, Division of Epidemiology and Prevention, Institute of Human Virology, University of Maryland School of Medicine, Baltimore, USA; i Healthy Sunrise Foundation, Las Vegas, USA; j Faculty of Medical Sciences and Dentistry, Department of Paediatrics and Child Health, University of Nigeria, Enugu, Nigeria; k Division of Epidemiology and Prevention, Department of Paediatrics and Faculty, Institute of Human Virology, University of Maryland School of Medicine, Baltimore, USA

**Keywords:** Nigeria, HIV, adherence, Option B plus, PMTCT, rural

## Abstract

The acceptability of lifelong antiretroviral therapy (ART) among HIV-positive women in high-burden Nigeria, is not well-known. We explored readiness of users and providers of prevention of mother-to-child transmission of HIV (PMTCT) services to accept lifelong ART -before Option B plus was implemented in Nigeria. We conducted 142 key informant interviews among 100 PMTCT users (25 pregnant-newly-diagnosed, 26 pregnant-in-care, 28 lost-to-follow-up (LTFU) and 21 postpartum women living with HIV) and 42 PMTCT providers in rural North-Central Nigeria. Qualitative data were manually analyzed via Grounded Theory. PMTCT users had mixed views about lifelong ART, strongly influenced by motivation to prevent infant HIV and by presence or absence of maternal illness. Newly-diagnosed women were most enthusiastic about lifelong ART, however postpartum and LTFU women expressed conditionalities for acceptance and adherence, including minimal ART side effects and potentially serious maternal illness. Providers corroborated user findings, identifying the postpartum period as problematic for lifelong ART acceptability/adherence. Option B plus scale-up in Nigeria will require proactively addressing PMTCT user fears about ART side effects, and continuous education on long-term maternal and infant benefits. Structural barriers such as the availability of trained providers, long clinic wait times and patient access to ART should also be addressed.

## Introduction

Nigeria is a key target in the global elimination of Mother-to-Child Transmission of HIV (eMTCT) agenda. Nearly 180,000 HIV-positive pregnant Nigerian women require PMTCT services every year (Nigeria National Agency for the Control of AIDS (NACA), 2016; UNICEF, 2018) however, only 32% of these women receive antiretroviral drugs (ARVs) for the Prevention of Mother-to-Child Transmission of HIV (PMTCT) (UNAIDS, 2017). This low maternal ARV coverage contributes to an estimated 36,000 Nigerian children acquiring HIV infection annually (UNAIDS, 2018). At 220,000, Nigeria has the highest number of children living with HIV (UNAIDS, 2018), and is one of 21 high-HIV burden African countries with the slowest decline (21%) in new HIV infections among children (UNAIDS, 2016).

Utilisation of maternal healthcare services is especially low in rural areas, where access to healthcare services are typically low, contributing to missed opportunities for averting new child HIV infections. The most recent national demographic survey reports only 47% of women receiving antenatal care (ANC) from a skilled provider and only 22% delivering at healthcare facilities (National Population Commission, 2013). PMTCT ARV coverage gaps are high in these rural communities as well (Federal Ministry of Health Nigeria, 2010a). Nigeria’s response to these PMTCT service gaps includes intensified PMTCT scale-up in rural communities, where services are largely provided by Primary Healthcare Centers (PHCs) (Federal Ministry of Health Nigeria, 2010b). PHC-based healthcare workers have accordingly been trained to provide PMTCT services in compliance with up-to-date guidelines.

Prior to 2010, the Nigerian national PMTCT guidelines recommended the World Health Organization (WHO) PMTCT Option A regimen which consisted of maternal zidovudine (AZT) during pregnancy and the short-term administration of single-dose nevirapine (NVP), azidothymidine (AZT), and lamivudine (3TC) at delivery and again, for one week postpartum. Breastfeeding infants were to receive daily NVP until one week after cessation of exposure to breast milk; for infants receiving formula, NVP was to be administered for only 6 weeks post-partum (Federal Ministry of Health Nigeria, 2007). In 2010, the revised national guidelines recommended at least three ARVs for pregnant women in compliance with WHO Option B (Federal Ministry of Health Nigeria, 2010a; World Health Organization, 2010). This involved continuing postpartum maternal ART until 1 week after cessation of breastfeeding for women who did not need ART for their own health. However, facilities such as PHCs, with low technical capacity for implementing Option B, were to continue implementing Option A for their clients (World Health Organization, 2010). The 2014 guidelines maintained the recommendation of Option B for PMTCT clients, expanding this to all healthcare facility levels (Federal Ministry of Health Nigeria, 2014). This was in contrast to the updated WHO-recommendation for life-long ART for all HIV-infected women after delivery (Option B+) (World Health Organization, 2013). Lifelong ART is expected to facilitate greater PMTCT coverage, minimise gaps in ARV prophylaxis between multiple pregnancies, reduce missed PMTCT opportunities, and reduce transmission among sero-discordant partners (World Health Organization, 2013).

Malawi, a south-Eastern African country with a current population of 19.1 million (Population Reference Bureau, 2018) and HIV burden of 1.0 million (UNAIDS, 2018) was the first country to implement Option B+. Malawi served as the litmus test for the effectiveness of Option B+. Subsequent reports noted impressive improvements in PMTCT ARV coverage and retention in care (Coutsoudis et al., 2013; Kalua et al., 2017; Tenthani et al., 2014). At 196 million people, Nigeria is the most populous country in Africa, accounting for over half (51.3%) of the population of West Africa (Population Reference Bureau, 2018), and has 3.1 million people living with HIV, the second-highest globally (UNAIDS, 2018). While there have been studies conducted on the acceptability, implementation, and impact of Option B+ in sub-Saharan African countries to date, these studies are lacking in West Africa in general, and in Nigeria in particular (Altan et al., 2016; Centre for Disease Control, 2013; Chimbwandira et al., 2013; Katirayi et al., 2016; Llenas-Garcia et al., 2016; Matheson et al., 2015; Ngarina et al., 2014). A study in Mozambique reported poor retention (∼50%) characterised by early loss-to-follow-up (LTFU) among women in rural areas (Llenas-Garcia et al., 2016). Given the lack of published studies on the acceptability of Option B+ in West Africa, this paper explores the perspectives of PMTCT users and providers on the readiness of HIV-positive women in rural Nigeria to accept and adhere to lifelong ART under Option B+.

## Materials and methods

### Study design, setting and population

This was a cross-sectional qualitative study conducted in 2014, employing semi-structured key informant interviews (KIIs) among women living with HIV enrolled in PMTCT programmes (‘Users’) and PMTCT service delivery personnel (‘Providers’). Providers were healthcare workers stationed at study PHCs who delivered clinical, counseling and drug dispensing services to Users.

The study was conducted at PHCs located in semi-rural and rural communities in two high-burden states in North Central Nigeria. The Federal Capital Territory (FCT) and Nasarawa State had high antenatal HIV prevalence of 5.8% and 6.3%, respectively, ranking above the national average of 3.0% (Federal Ministry of Health Nigeria, 2010b). HIV services at the study PHCs are supported by the Institute of Human Virology-Nigeria (IHVN), a large public health non-governmental organisation that is an implementing partner of the United States government’s President’s Emergency Plan for AIDS Relief (PEPFAR). At the time of the study, IHVN was providing support to nearly 1,500 primary, secondary and tertiary health facilities in 10 states, including the FCT and Nasarawa state. In these locations, IHVN was the single largest PEPFAR implementing partner providing HIV services, including HIV testing, PMTCT, ART and TB/HIV services. All women enrolled in PMTCT programmes at the time of the study (2014) were prescribed Option B ART as recommended by the then-national guidelines, in line with WHO recommendations (Federal Ministry of Health Nigeria, 2014; World Health Organization, 2013).

### Participant recruitment (PMTCT users and providers)

All Users to be interviewed were HIV-infected women ≥18 years old living in the PHC catchment area and accessing care from the study PHC. As we envisaged interviewing women at key points of the PMTCT cascade, we recruited Users based on the following criteria:

- Currently pregnant and in care (established on ART and in PMTCT care);
- Postpartum (within 3 months post-delivery and breastfeeding) and in PMTCT care;
- Newly HIV-diagnosed (within 7 days) and not yet enrolled in care;
- Postpartum and classified as lost to follow-up (LTFU) from the PMTCT programme (≥3 months without a PMTCT service accessed).

Purposive sampling was employed in selecting these respondents because women in rural areas, especially HIV-positive women, have low rates of facility-based healthcare service utilisation (Federal Ministry of Health Nigeria, 2012; National Bureau of Statistics, 2015). Eligible women attending clinic were identified and briefed about the study by healthcare workers at the study sites. Interested clients were then individually approached by study staff for informed consent. LTFU women were also identified from medical registers and then were contacted by phone by healthcare workers; those interested were then invited to the PHC for the interview. Home interviews were also arranged for LTFU women who were interested in participating in the study but declined to interview at the facility. PMTCT providers at study sites were recruited to contribute their perspectives, based on their experiences, on Option B attitudes and practices, for their opinions on Option B+ acceptability among Users. Providers recruited included doctors, pharmacists or pharmacy technicians, nurses, and community health workers providing direct clinical services to PMTCT clients.

During orientation to the study and consent process, researchers introduced themselves, stating where they worked (with an NGO/university and not the health facility or government), and the reason for conducting the study, namely, to improve the quality of health services for women living with HIV. Written informed consent was obtained and study interviews conducted only by trained research staff who had neither an affiliation with the healthcare facility nor an affiliation with IHVN’s PMTCT programme. Data was collected in private rooms of health care facilities during non-clinic hours. While we did not collect information on the reasons why, less than 10% of participants who were approached declined to participate. No one else was present during these interviews besides participants and researchers.

### Study questionnaires and KII implementation

Multi-theme, semi-structured questionnaires were developed and presented to physicians, nurses, public health specialists and social/behavioural researchers (Table 1). The questionnaire was modified to suit the clinical and cultural context. KII guides were developed by the authors, taking care to pose open-ended questions that were amenable to reframing by facilitators if participants did not understand them, regardless of language used. Questions on highly sensitive issues eg self-stigma, poor adherence and poor retention were posed as third person questions (ie ‘they’; not ‘you’) in order to allow participants to freely express their opinions without ‘implicating’ themselves. The guides were adjusted as interviews were conducted, per emerging data and recurring questions. Users and providers were interviewed only once for this study. The Users KII questionnaire contained open-ended questions organised under four specific themes (Table 1).

**Table 1.** Major study questions and themes.

Themes	Themes
*User Questionnaires*	
Knowledge and perceptions on MTCT and breastfeeding	Knowledge and perceptions on MTCT and breastfeeding
Perceptions regarding maternal treatment and drug adherence in PMTCT	Perceptions regarding maternal treatment and drug adherence in PMTCT
Reasons for poor Option B drug adherence	Reasons for poor Option B drug adherence
Perceptions regarding changing from Option B to Option B+ practice	Perceptions regarding changing from Option B to Option B+ practice
Provider Questionnaires	
Factors affecting current Option B drug adherence among PMTCT clients	Factors affecting current Option B drug adherence among PMTCT clients
Perceptions on Option B+ readiness and acceptability among PMTCT clients	Perceptions on Option B+ readiness and acceptability among PMTCT clients

During these interviews, PMTCT user perceptions on the acceptability and feasibility of changing practice from Option B to Option B+ were elucidated. For the Providers, we explored their perspectives regarding Option B+ acceptability and readiness among PMTCT clients. Each KII was facilitated by two trained research staff: one posed questions and the other took notes. All interviews were digitally recorded, and each recorded KII was transcribed verbatim. Each KII took an average of 45 minutes to one hour to complete. For participants who did not speak English, KIIs were conducted in the dominant local language Hausa by skilled bilingual study staff, who then transcribed and translated the local-language interviews into English.

### Data analysis

Data transcription, translation, review and preliminary analysis started in conjunction with data collection. All KIIs were transcribed by the same study staff who conducted the interviews. For the manual qualitative analysis, we adopted the constant comparative method in a grounded theory approach (Glaser & Strauss, 2009). In this approach, inductive methodology is used to systematically generate theory from the data collected. Analysts read transcripts multiple times to become familiar with the data, identify patterns and generate initial codes. Following this initial analysis, emerging content-driven themes and sub-themes were discussed, codes refined and categories developed independently by a panel of eight paired researchers. Finally, all researchers collaborated in triangulation as a means of verification of our findings as well as to eliminate any biases that may have occurred during individual analysis. Quantitative demographics data was analysed with Statistical Package for Social Sciences (SPSS V.16.0) for Windows.

*Ethical Considerations:* This study was approved by the Nigerian National Health Research Ethics Committee and the Institutional Review Board of the University of Maryland Baltimore.

## Results

A total of 142 participants were interviewed, comprising of 100 PMTCT Users (Table 2) and 42 PMTCT Providers (Table 3). Most of the PMTCT users were between the ages of 21 and 30 (69%), married (86%), had between one and four children (74%) and had a least a secondary education (64%) (Table 2).

**Table 2.** PMTCT user demographics.

Characteristics	Pregnant, in care *N* (%)	Postpartum, breastfeeding *N* (%)	Newly-diagnosed, pregnant *N* (%)	Lost to follow-up *N* (%)	Total, *N* (%)
Age group (years)	*n* = 27	*n* = 27	*n* = 24	*n* = 22	*n* = 100
< 21	0 (0.0)	0 (0.0)	4 (16.7)	3 (13.6)	7 (7.0)
21–30	16 (59.3)	22 (81.5)	18 (75.0)	13 (59.1)	69 (69.0)
31–40	11 (40.7)	5 (18.5)	2 (8.3)	6 (27.3)	24 (24.0)
Marital status	*n* = 27	*n* = 27	*n* = 24	*n* = 22	*n* = 100
Single	1 (3.7)	1 (3.7)	0 (0)	3 (13.6)	5 (5.0)
Married	24 (88.9)	24 (88.9)	24 (100)	14 (63.6)	86 (86.0)
Widowed	2 (7.4)	2 (7.4)	0 (0.0)	2 (9.1)	6 (6.0)
Divorced	0 (0.0)	0 (0.0)	0 (0.0)	3 (13.6)	3 (3.0)
Number of children	*n* = 27	*n* = 26	*n* = 24	*n* = 21	*n* = 98
0	7 (25.9)	1 (3.8)	7 (29.2)	4 (19.0)	19 (19.4)
1–2	14 (51.9)	18 (69.2)	9 (37.5)	5 (23.8)	46 (46.9)
3–4	5 (18.5)	6 (23.1)	6 (25.0)	9 (42.9)	26 (26.5)
≥ 5	1 (3.7)	1 (3.8)	2 (8.3)	3 (14.3)	7 (7.1)
Educational Attainment	*n* = 27	*n* = 26	*n* = 24	*n* = 22	*n* = 99
None	4 (14.8)	0 (0.0)	2 (8.3)	5 (22.7)	11 (11.1)
Primary	6 (22.3)	7 (26.9)	8 (33.3)	6 (27.3)	27 (27.3)
Secondary	13 (48.1)	16 (61.6)	9 (37.5)	9 (40.9)	47 (47.5)
Post-secondary	4(14.8)	3 (11.5)	5 (20.8)	2 (9.1)	14 (14.1)

**Table 3.** PMTCT provider demographics.

Characteristics	Doctors *N* (%)	Pharmacy Staff *N* (%)	Nurses/ Community Health Workers *N* (%)	Adherence Counsellors *N* (%)	Total *N* (%)
Gender	*n* = 10	*n* = 13	*n* = 11	*n* = 8	*n* = 42
Male	9 (90.0)	5 (38.5)	1 (9.1)	0 (0.0)	15 (35.7)
Female	1 (10.0)	8 (61.5)	10 (90.9)	8 (100.0)	27 (64.3)
Age group (years)	*n* = 10	*n* = 13	*n* = 11	*n* = 8	*n* = 42
21–30	2 (20.0)	1 (7.7)	0 (0.0)	2 (25.0)	5 (11.9)
31–40	6 (60.0)	6 (46.2)	6 (54.5)	4 (50.0)	22 (52.4)
41–50	2 (20.0)	4 (30.8)	4(36.4)	2 (25.0)	12 (28.6)
51 and above	0 (0.0)	2 (15.4)	1 (9.1)	0 (0.0)	3 (7.1)
Marital Status	*n* = 10	*n* = 13	*n* = 11	*n* = 8	*n* = 42
Single	3 (30.0)	0 (0.0)	0 (0.0)	1 (12.5)	4 (9.5)
Married	7 (70.0)	13 (100.0)	11 (100.0)	7 (87.5)	38 (90.5)
Education	*n* = 10	*n* = 13	*n* = 11	*n* = 8	*n* = 42
Secondary	0 (0.0)	0 (0.0)	0 (0.0)	1 (12.5)	1 (2.4)
Post-Secondary	0 (0.0)	11 (84.6)	6 (54.5)	5 (62.5)	22 (52.4)
Tertiary/Higher	10 (100.0)	2 (15.4)	5 (45.5)	2 (25.0)	19 (45.2)

Among the PMTCT users, 22% were lost to follow-up (LTFU), with a disproportionately higher percent of that sub-population representing women with three to four children (43%) or with secondary education (41%). The patients in this study were seen by predominately female providers (64%) (Table 3). Within the sub group of PMTCT providers, 90% of the physicians were male, while over 60% of the pharmacists/pharmacy technicians, nurses/community health workers and adherence counsellors were female. The majority (78%) of the PMTCT providers were between the ages of 31 and 50 years, and 90% were married.

### Pmtct users’ opinions regarding lifelong ART (Option B+)

While women across all four sub-groups found ART during pregnancy and breastfeeding acceptable for prevention of infant HIV, there were mixed views regarding lifelong ART. Some women expressed motivation to continue: ‘*Yes I shall be taking the drugs always. You know HIV is not an instant sickness, it is a gradual step. I need to be taking my drugs so that it will not rise again’* (Pregnant, in care). ‘*I will have to continue for my safety so I can live long and take care of my children’* (Newly-diagnosed, pregnant). Others had reservations about long-term ART: ‘*No, I would not continue taking my drugs after delivery, not if my CD4 count is high’* (Pregnant, in care). *‘I might stop taking the drugs at that point for a while then when my CD4 drops again, I shall continue taking the medicine’* (Postpartum, breastfeeding). ‘*I am ok, there is no need for drugs for now’* (LTFU).

Within the sub groups of PMTCT users, most pregnant in-care women, and those LTFU consistently supported lifelong ART and adherence. One pregnant in care respondent stated: ‘*I can’t stop taking my drugs, so far that I am still alive.’* Women who were LTFU were more likely to accept lifelong ART, albeit with some conditions: ‘*I want to take the drugs so that I can be normal and I won't be sick.’ ‘If it* (non-adherence) *won’t cause me to be sick, I won’t take the drugs, honestly, but if they say there will be a problem I will continue taking it.* Pregnant women who were newly HIV-diagnosed expressed willingness to take ART for life: ‘*I shall continue taking the drugs regardless of my CD4 count’.* Another woman indicated: *‘Yes sir. I shall use my drugs for life … so that it will reduce the sickness in me and I will not be sick always’*.

On the other hand, the responses of post-partum mothers suggested they had some problems with the idea of continuing ARVs after delivery, especially when all HIV exposure to the infant has ceased and maternal anxiety regarding MTCT is abated: ‘*It is easiest to take the drugs while pregnant because I want the medicine to start working quickly and to reduce the virus in my body so I don't transmit it to my child’.* A second mother indicated that *‘It is because of my baby that I am taking it* (ARVs).’ At the same time, across all groups*,* PMTCT users indicated that non-adherent women have multiple reasons for non-adherence to medications which include drug side effects, ‘*Maybe some don’t like the smell; some say it makes them sick, that’s why they don’t take the drugs’* (Post-partum, breastfeeding). This was reinforced by the experiences of several LTFU women: ‘*If I take it I get dizzy and my stomach aches; I will have to get Maltina (malt drink) or milk before I feel better, so any time I don’t have money for that, I leave the drugs’*.

I stopped taking ART because whenever I take it, I vomit so seriously. Whenever I take the drugs, it makes me stool seriously.

Other reasons for non-adherence were stigma, staff attitude, hopelessness and status denial as reflected in the following quotes: ‘*Because they don’t want other people to know that they are infected’ (*Pregnant, in care). *They feel shy to go and collect their drugs’* (Post-partum, breastfeeding), ‘*This stigmatization problem, the way those health people handle us is not good, but if you are well taken care of and everything is confidential, it is better’* (LTFU). ‘*They think since they are already infected the next thing is death’* (Pregnant, newly diagnosed)

### PMTCT provider opinions regarding lifelong ART (Option B+)

PMTCT Provider interview responses focused primarily on the challenges they saw in clients being compliant with current Option B procedures that could impact on Option B+ uptake and success. All PMTCT Provider sub-groups uniformly identified users’ lack of education and poor understanding of the goals and expected outcomes of PMTCT. They also thought that these differences varied by the background of the user. *‘We have seen some mothers who are positive but not pregnant, who refuse drugs … most of them who refuse drugs on their own, when it comes to the pregnancy most take them.’* ‘*Some of them are not adequately informed on the benefits of the drugs so they take it at will. They should be adequately informed to take the drugs at the right time … ’* According to an adherence counsellor:

Our community is a village. Those (women) that are educated understand (the importance of adherence) easily. Those that are not educated usually refuse because their husbands do not allow them to take it. Sometimes it depends on the acceptance of the husband.

Consistently, PMTCT providers observed that the post-delivery period appeared to be a risky time in the PMTCT cascade to introduce the concept of lifelong ART to the HIV-infected women. One doctor stated

We have seen some mothers who are positive but not pregnant, who refuse drugs. But I will say that most of the time, for most of them who refuse drugs on their own, when it comes to the pregnancy most take them.

Another doctor supported this finding: *‘They are open to it* (ART) *during pregnancy, but after delivery they refuse to take the drugs.’* Illness or the threat of illness was also a motivator for drug uptake: ‘*Fear* (of illness) *makes them take their drugs. They also take it to keep their children safe.’*

During these interviews, PMTCT providers indicated that education regarding drug regimens and adherence was central to the successful introduction of Option B+. In particular, the they suggested that the rolling out of gradual education strategies regarding drug adherence; the possible side effects of ART; and how patients can manage their care while staying on ART would be productive in the future. One nurse indicated that some patients ‘*complain of side effects, most especially vomiting, dizziness. These are the common side effects.’* On the other hand, ‘*most of them, when you ask, they will tell you there are no side effects and no danger taking the drugs, as far as it will treat them for what is needed, they don’t feel danger in taking ART.’*

Within the treatment delivery process, a nurse presented the concept of a gradual introduction of long term ART by stating that.

I start by playing with them and then move to the reality … I ask if the patient can take drugs for a week, then two weeks, then a month. I say this gradually until the patient accepts. I tell them that they need to take the drugs to suppress the HIV cells and let the CD4 count rise, I tell them that they can marry and have HIV negative children and live a long life.

Along the same lines a pharmacist stated: *‘Some of these ladies are not used to taking drugs for long periods. We have to encourage them, talk to them, and tell them the implications not only to them but the baby we want to save.’* Finally, for the adherence counsellors, they reported that fostering acceptability to Option B+ became a balancing act of addressing the worries and concerns of the newly diagnosed client while encouraging the client to make their appointments and improving understanding how ART may benefit them.

## Discussion

Our study sheds light on the acceptability of lifelong ART among four different categories of women living with HIV in high HIV-burden rural communities in north-central Nigeria. PMTCT users had mixed views about the lifelong use of ART; pregnant in-care and LTFU women proposed conditional acceptability – in the event of threatened illness from non-adherence. Post-partum Users struggled to maintain motivation for continuing ART after delivery.

Conversely, newly-diagnosed Users expressed willingness to take ART for life. This suggests that Option B Plus is likely best initiated at HIV diagnosis before or during pregnancy, when motivation for preventing infant HIV may be high. This finding is corroborated by studies from East and southern Africa (McLean et al., 2017; Schnack et al., 2016) However, follow-up and retention even for these motivated women may be problematic if providers are not equipped to counsel and support clients during the latter stages of pregnancy, and between pregnancies. Overall, findings suggested that PMTCT Users would not be sufficiently prepared to start and sustain lifelong ART without concomitant long-term education, counselling and psychosocial support.

Studies conducted by our research team in Nigeria (Sam-Agudu, Cornelius, Okundaye, Adeyemi, Isah, Isah et al., 2014; Sam-Agudu, Cornelius, Okundaye, Adeyemi, Isah, Wiwa et al., 2014; Sam-Agudu, Ramadhani, Isah, Anaba, et al., 2017; Sam-Agudu, Ramadhani, Isah, Erekaha, et al., 2017) and by other researchers in Malawi (Cataldo et al., 2017; Phiri et al., 2017) suggest that mentor mothers can provide acceptable and impactful psychosocial support in PMTCT, whether women are on lifelong ART or not. Mentor Mothers are women living with HIV who have prior experience in successfully navigating the PMTCT cascade of care, and ideally have an HIV-negative child. These women are trained to provide pyschosocial support to other women enrolled in PMTCT programmes in order to achieve optimal PMTCT outcomes of maternal health and infant HIV-free survival through healthy behaviours including ART adherence, making appointments, disclosure to male partners, appropriate infant feeding, and positive living. Given their lived experiences with HIV and as PMTCT clients, mentor mothers can potentially improve acceptability of ART (lifelong or not) among other HIV-positive women. Additionally, mentor mothers can provide ongoing and longterm support for women who may be vulnerable to cessation of ART due to drug fatigue and de-motivation. Ultimately, it may be helpful for peer support to be provided shortly after engagement in lifelong ART programmes regradless of whether clients are newly- or previously on ART.

PMTCT Providers recognised the need for continuous patient education regarding the merits of lifelong ART. They also flagged the period after childbirth as a high-risk time for introducing the concept of lifelong ART to patients as they noticed a significant drop in patient visits after the birth of the child. The call for more structured PMTCT patient education during the postnatal period is echoed by other African studies (Nachega et al., 2012; Ngarina, Popenoe, Kilewo, Biberfeld, & Ekstrom, 2013; Ngarina et al., 2014). Given the complexities involved in the delivery of HIV care, especially in Nigeria, it appears that implementation of lifelong ART may require a stage-focused communication and education approach, which has been shown to be more effective than uniform health-promotion messages (Forthofer & Bryant, 2000; Kreuter & Wray, 2003; McDermott, 2000). Furthermore, PMTCT User population segmentation may be useful to inform approaches for social marketing programmes targeting behaviour change. Considerations of locally-derived results such as this study, coupled with a PMTCT stage-specific approach can guide Nigeria’s implementation of Option B Plus for maximal impact. That said, facility-based education/counselling alone may not be sufficient, and intensive follow-up in the community may be warranted-this can be performed by community health workers and/or mentor mothers.

Our findings of the fear of drug side effects and reluctance in committing to lifelong therapy in the absence of symptoms are also supported by Ngarina et al’s Tanzanian study (Ngarina et al., 2014). Besides the fear of side effects, fear of HIV disclosure to male partners, community-based HIV/AIDS stigma, discrimination, and negative health workers’ attitudes as concerns or barriers to the implementation of Option B+ (Elwell, 2016; Kruk et al., 2016; Marinda, Chibwe, Tambo, Lulanga, & Khayeka-Wandabwa, 2017).

The role of stigma in low ART acceptability and poor sustainability of adherence in our setting cannot be ignored. In rural, closed communities such as our study setting, women living with HIV may experience stigma (and discrimination), from their own partners, families, communities and even healthcare workers including PMTCT providers. Our team has documented these findings among Option B PMTCT clients in Nigeria (Cornelius, Erekaha, Okundaye, & Sam-Agudu, 2017; Odiachi et al., 2018), and again these women have found peer and community support useful for overcoming stigma-related barriers to sustaining treatment adherence.

Malawi’s early-adopted Option B plus programme offers countries with later Option B plus adoption some lessons learned for local application. Kim and colleagues (Kim et al., 2016), highlighted drivers for re-starting lifelong treatment by women who initially refused or stopped ART in Malawi. These included encouragement from community health/lay workers, side effects subsiding, decline in health, supportive male partner, and fear of future sickness. While the composition and delivery of strategies to facilitate these drivers may be different in our setting, our study supports the targeting of these drivers to mitigate the barriers our study participants have outlined. Ultimately, many of the barriers and facilitators relating to interrupted PMTCT ART (Option B) will apply to lifelong ART through Option B plus; the goal is to further strengthen and scale up evidence-based, locally-adapted strategies as Option B plus is scaled up in different settings.

### Study limitations

Our study has some limitations. First, we did not obtain information on gravidity of User participants; as such we were unable to assess the influence of gravidity and PMTCT stage on their opinions regarding lifelong ART. The study also did not collect information on User participants’ experience with stopping and starting ART due to multiple pregnancies under Option B. Lastly, this study would have been strengthened with a formal mixed-methods approach where characteristics of participants eg age, education, marital status, timing of diagnosis, years on ART could have been correlated with their categorisation into each of the four groups.

## Conclusions

By 2015, all Global Plan priority countries except Nigeria had adopted the Option B plus. Lack of local evidence, actors (interest and power) and context (low domestic funding and poor retention in care) affected the policymaking process (Olakunde & Ndukwe, 2017). Nigeria finally adopted Option B+ in its 2016 HIV treatment guidelines (Federal Ministry of Health Nigeria, 2016), in part due to the findings of a nationwide study that reported that when Option B and Option B plus are considered as treatment options for pregnant women living with HIV, the latter is more cost-effective (Adesina & Alkenbrack, 2015). Our study adds to this new and welcome Option B plus development for Nigeria, by highlighting that client motivations for acceptability and sustained adherence are likely influenced by stage and engagement (or not) in the PMTCT cascade. Women living with HIV are likely to have different motivations to accept and adhere to lifelong ART at different points of their engagement with PMTCT programmes, and our service delivery package should accommodate this finding, by incorporating long-term peer support, provider education, and staged/timed intensification of education and counselling when it is most needed.

## Data Availability

The datasets generated and/or analysed during the current study are not publicly available due to ongoing analysis for future publication but are available from the corresponding author upon reasonable request.
